# IT’S GRATEFULNESS: THE ROLE OF GRATITUDE ON ADVERSE OUTCOMES AMONG BEREAVED BLACK ADULTS

**DOI:** 10.1093/geroni/igad104.1666

**Published:** 2023-12-21

**Authors:** Danielle McDuffie

**Affiliations:** Durham VAMC, Durham, North Carolina, United States

## Abstract

The Black population has been found to experience more bereavement than other racial/ethnic groups. Accompanying loss for Black populations is often other stressful factors such as racial trauma, childhood trauma, and comorbid depression. However, despite these hardships, one of the most prevalent strengths in the Black population is their engagement in religious faith as a means of coping. Gratitude, a major factor in Positive Psychology, shares many tenets with religious beliefs; however, the utility of gratitude has yet to be fully investigated within a Black bereaved population. This study sought to identify whether gratitude moderated the relationship between experienced negative life events (i.e., racial trauma; RT, adverse childhood experiences; ACEs) and depression in a sample of bereaved Black middle to older adults. 103 Black adults aged 45+ (33% female) were administered items assessing depression, gratitude, RT, and ACEs as part of a larger bereavement study. ACEs and RT both significantly predicted depression (p=.009, p=.008, respectively), and there was a significant conditional effect of gratitude on depression, reflecting differences in the endorsement of depression across levels of ACEs and RT. Accordingly, with the significant role of gratitude amongst the present sample, there is evidence for utilizing a strengths-based, Positive Psychology informed approach in the clinical care of Black adults experiencing bereavement, particularly in the context of impactful and racially traumatic events. Additionally, a fruitful direction for future research and policy is addressing the role of adverse traumatic events on Black bereavement.

